# The Usage of Digital Health Technology Among Older Adults in Hong Kong and the Role of Technology Readiness and eHealth Literacy: Path Analysis

**DOI:** 10.2196/41915

**Published:** 2023-04-12

**Authors:** Seungmo Kim, Bik Chu Chow, Sanghyun Park, Huaxuan Liu

**Affiliations:** 1 Department of Sport, Physical Education, and Health Hong Kong Baptist University Hong Kong China (Hong Kong); 2 Department of Sport for All Korea National Open University Seoul Republic of Korea; 3 School of Physical Education and Sport Science Fujian Normal University Fujian China

**Keywords:** older adults, elderly, digital health technology, health technology, digital health, technology readiness, eHealth literacy, continuous usage intention, usage intention, intention-to-use, attitude, technology use, elder, digital literacy, adoption, acceptance, readiness, gerontology, aging

## Abstract

**Background:**

Although digital health technologies (DHTs) help many people maintain a healthy life, including those of advanced age, these technologies are of little use to older adult populations if they are not being adopted in daily life. Thus, it is critical to identify ways to help older adults recognize and try new technologies and maintain their use of them to maximize the benefits of these technologies in a digital-based society.

**Objective:**

Our study aimed (1) to assess the current usage of DHT among older adults in Hong Kong and (2) to examine how high and low levels of eHealth literacy in this group affects the relationship between the Technology Readiness and Acceptance Model (TRAM) and attitudes and intention toward DHT.

**Methods:**

A total of 306 adults over 60 years of age in Hong Kong participated in this study. After conducting confirmatory factor analysis to validate the measurement model, the hypothesized model was tested using structural equation modeling.

**Results:**

Optimism was significantly related to perceived usefulness, while optimism, innovativeness, and discomfort were significantly associated with perceived ease of use. Both perceived usefulness and perceived ease of use were significantly linked to attitude toward the use of DHTs. Meanwhile, attitude significantly predicted usage intention. Additionally, the results revealed the differences in the relationships of the TRAM between participants with high and low levels of eHealth literacy. The influence of optimism and innovativeness on perceived ease of use was stronger for the higher-level group than for the lower-level group, and the influence of discomfort for the higher-level group was much weaker.

**Conclusions:**

The findings provided partial support for the impact of eHealth literacy on encouraging older adults to use DHT and obtain health benefits from it. This study also suggests providing assistance and guidelines for older adults to narrow the aging-related technology gap and to further explore the associations of eHealth literacy, the TRAM, and actual behaviors.

## Introduction

### Background

Rapid advances in medical science and technology have made it possible to detect diseases much earlier and provide appropriate treatment for those that were previously considered incurable. These advances have also enabled a variety of advanced health-related services and treatment techniques to be received more comfortably and effectively, which may lead to increased life expectancy [1]. According to the recent census in Hong Kong [2], in 2021, the proportion of adults aged 65 years and above was 20% of the total population, an increase of 7% over the past 10 years, and it is expected to increase continuously. Hong Kong, one of the fastest aging societies in the world, considers health and care services for older adults an important social issue [3].

Among various technologies to help people maintain a healthy life, digital health technology (DHT), which applies digital transformation technology to the health care field and includes mobile health (mHealth) apps, wearable devices, electronic health records, and electronic medical records, is an innovative and efficient means to offer people a healthier life, particularly for older populations [4]. For instance, mHealth apps make it easy for older adults to schedule medical appointments and collect and archive health data and records, and they allow medical and health care staff to continuously monitor their patients and improve patient‒doctor communication [5].

Although DHT is presented as an important way to achieve a healthy life and new technologies have been designed and developed to provide a better quality of life for older individuals, these technologies are purposeless unless older adults use them. Therefore, identifying ways to help older people recognize and try new technologies and maintain their use of these technologies is critical to enable them to benefit from these technologies in a digital-based society. In the last 3 decades, many studies based on various theoretical models and theories have been conducted to understand older adults' intention to use these technologies and to identify relevant precedents [6]. These models have proposed and tested different antecedents to understand their effect on users’ acceptance of DHTs. These theories include the Technology Acceptance Model (TAM) [7], the unified theory of acceptance and use of technology [8], the social cognitive theory [9,10], and the theory of planned behavior. Many studies [4] that use these theories have studied the antecedents of the technology adoption behaviors of older adults. The antecedents include technology factors (eg, perceptions of usefulness and ease of use, performance expectancy, and effort expectancy), psychological factors (eg, self-efficacy, technology anxiety/anxiety, attitude, and hedonic motivation), social factors (eg, social influence and subjective norms), personal factors (age, education, and gender), environmental factors (eg, facilitating conditions), and price value [4].

Among the technology adoption models, the TAM has been the most frequently used to understand people’s information technology adoption behaviors in the health care context as well as in other fields. The TAM, developed by [7] based on the theory of reasoned action [11] as a framework to explain people’s adoption of information technology in their work, assumes that levels of attitude toward the use of and intention to use technology may depend on users’ perceived usefulness (PU) and perceived ease of use (PEU) for the technology. Davis [7] defined PU as “the degree to which a person believes that using a particular system would enhance his or her job performance” and PEU as “the degree to which a person believes that using a particular system would be free of effort.” In this model, PEU should influence PU.

As mentioned, the TAM has been empirically replicated to explain people’s behaviors with regard to adopting technologies in various fields, such as marketing, education, banking, social media, and health care [12,13], and has been extended based on the belief that PEU and PU should be influenced by external variables. The Technology Readiness and Acceptance Model (TRAM), which this study uses, is one of the extended models of the TAM. In addition, this study incorporates eHealth literacy, which reflects individuals’ ability to use novel information and communication technology, especially the internet, to improve or enable their health and health care [14], to examine the impact of eHealth literacy on the relationships proposed by the TRAM. eHealth literacy is considered an important factor in reducing multiple access barriers to digital technology, which can increase health literacy and help older adults develop deeper knowledge and better self-care [15]. Therefore, the main purposes of this study are (1) to assess the current usage of DHTs among older adults in Hong Kong, (2) to explore the factors influencing usage intention, and (3) to examine how the eHealth literacy of older adults affects this relationship.

### Theoretical Foundations

**Figure 1:**
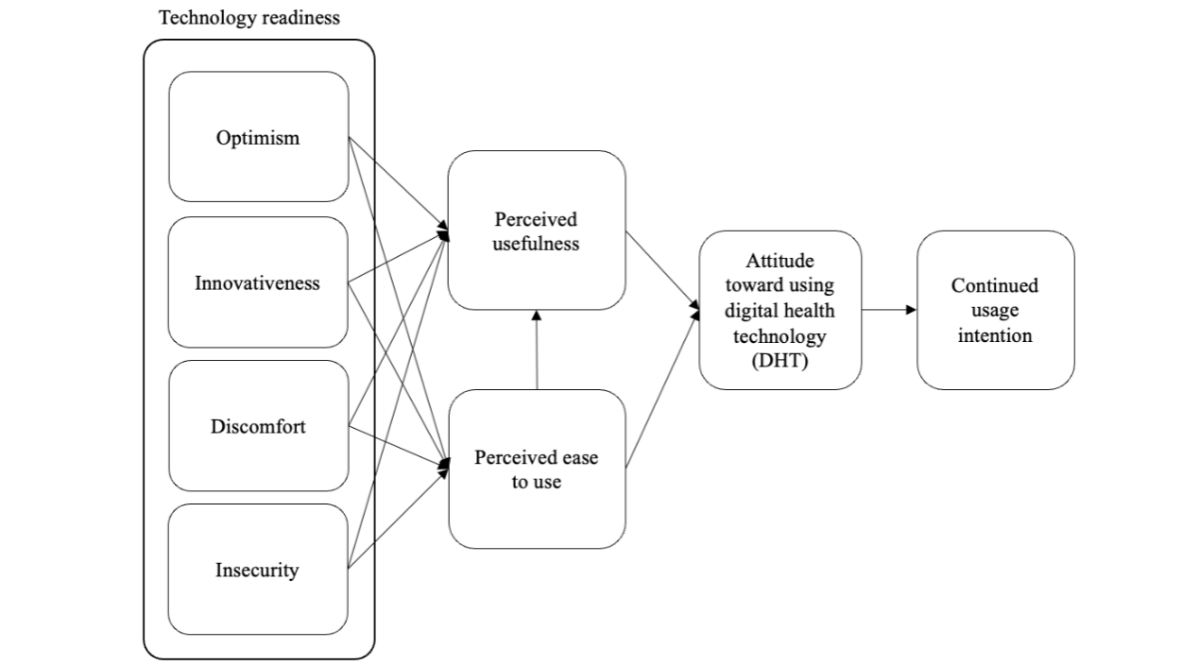
Research model.

As shown in Figure 1, the proposed conceptual model consists of five constructs: (1) technology readiness (TR) (eg, optimism, innovativeness, discomfort, and insecurity), (2) PU, (3) PEU, (4) attitude toward using technology, and (5) continued usage intention. In this model, eHealth literacy is expected to moderate the proposed relationships.

As one of the extended models of the TAM, the TRAM incorporates TR and people’s propensity to accept and use new technologies to achieve their goals at home and work [16]. TR is an overall mental state resulting from a gestalt of mental activators and inhibitors that collectively determine an individual’s tendency to use new technologies. TR consists of four subdimensions: (1) optimism, an individual’s positive view of technology and the belief that technology provides people with improved control, flexibility, and efficiency; (2) innovativeness, an individual’s tendency to become a technology pioneer and thought leader; (3) discomfort, which is the feeling of being overwhelmed by technology and a perception of a lack of control over technology; and (4) insecurity, including distrust of technology and skepticism about how it performs. Among the 4 dimensions of TR, optimism and innovativeness act as driving forces, while discomfort and insecurity are inhibitory factors. Many studies have confirmed the relationships between TR and variables such as PU, PEU, and attitude and intention in the TAM. For instance, customers with a high level of TR had a higher perception of the usefulness of technology, which also positively influenced their behavioral intentions [17,18]. Liljander et al [19] found that the driving forces of TR, such as optimism and innovativeness, were associated with the perception of the ease of use of new technology and weakened the impact of the perception of the usefulness of the technology on behavioral intention. In other words, people with high levels of optimism and innovativeness are likely to take risks and tolerate uncertainties related to new technology, while inhibitory factors such as insecurity and discomfort lower perceptions of usefulness and the ease of use of new technology [20,21].

Previous literature on technology acceptance in health care and services has studied various individual characteristics and external variables that could affect the relationships proposed by the TAM, but no research has incorporated TR along with PU and PEU on attitudes and intentions. The conceptual model (Figure 1) and hypotheses have been proposed based on previous research on the TRAM and these variables. The proposed seven hypotheses are as follows:

- Hypothesis 1: TR (H1-a: optimism, H1-b: innovativeness, H1-c: discomfort, and H1-d: insecurity) influences PU.
- Hypothesis 2: TR (H2-a: optimism, H2-b: innovativeness, H2-c: discomfort, and H2-d: insecurity) influences PEU.
- Hypothesis 3: PEU influences PU.
- Hypothesis 4: PU influences attitudes toward the use of DHT.
- Hypothesis 5: PEU influences attitudes toward the use of DHT.
- Hypothesis 6: Attitudes toward the use of DHT influence continued usage intention.
- Hypothesis 7: The level of eHealth literacy will influence the relationships between TR and the variables.

## Methods

### Participants

The population of this study comprised adults over 60 years of age in Hong Kong. A web-based survey method was used to collect data in this study via a convenience sampling method. A total of 357 participants completed the survey, of whom 306 provided usable responses. The demographic characteristics are shown in Table 1. To identify specific characteristics of eHealth literacy, we conducted a crosstab analysis by dividing the sample into 2 groups using the average (mean 3.417, SD 0.805) of eHealth literacy. Among the 5 variables, there was a significant difference between the groups with regard to marital status (*χ*^2^_3_=10.180; *P*=.006) and perceived socioeconomic status (*χ*^2^_4_=12.240; *P*=.02). The proportion of unmarried people included in the low eHealth literacy group (group A) tended to be high. Additionally, it was found that the higher people’s perceived socioeconomic status was, the more likely they were to be included in the high eHealth literacy group (group B).

Table 2 shows detailed information about the participants’ internet usage. With regard to average internet use a day, 31% (n=92) of participants used the internet for 2 hours, and 18.5% (n=53) of participants used the internet for 3 hours a day. Hence, approximately 50% (n=145) of participants use the internet for 2-3 hours a day. Additionally, participants who used the internet for equal to or greater than 5 hours a day accounted for approximately 23% (n=71). In the case of access type, the majority were mobile (91.5%, n=280). The most represented web-based activities using the internet were as follows: games (74%, n=227), banking or finance transactions (63%, n=192), searching for health information (50%, n=152), chatting (47%, n=144), and office or personal affairs (44%, n=134).

**Table 1:** Crosstab analysis for demographic characteristics.

Characteristics			Total, N		Group A (n=141), n		Group B (n=165), n		*χ*^2^ (*df*)			*P* value	
**Gender**										0.935 (1)			.33
	Male	113		48		65							
	Female	193		93		100							
**Age (years)**										5.602 (2)			.06
	55-59	65		25		40							
	60-65	154		67		87							
	66 and older	87		49		38							
**Marital status**										10.180 (2)			.006
	Single	46		30		16							
	Married	230		102		128							
	Divorced or widowed	30		9		21							
**Educational level**										4.162 (5)			.53
	Elementary	10		7		3							
	High school	68		30		38							
	College degree	56		25		31							
	Junior high	33		18		15							
	Certificate	57		23		34							
	Graduate degree	82		38		44							
**Perceived socioeconomic status**										12.240 (4)			.02
	Very low	9		6		3							
	Low	53		32		21							
	Medium	190		87		103							
	High	48		15		33							
	Very high	6		1		5							

**Table 2:** Information about participants’ internet usage.

Variables		Participants, n (%)
**Average internet use per day**		
	1 hour	43 (14)
	2 hours	92 (31)
	3 hours	53 (18)
	4 hours	39 (13)
	≥5 hours	71 (23)
**Access type^a^**		
	Mobile	280 (91)
	PC	182 (59)
	PC, somewhere else	38 (12)
	Other	12 (4)
**Web-based activity^a^**		
	Chatting	144 (47)
	Reading news	81 (26)
	Entertainment	109 (36)
	Games	227 (74)
	Shopping	87 (28)
	Searching health information	152 (50)
	Office/personal affairs	134 (44)
	Bank/finance transaction	192 (63)

^a^Participants were able to choose more than 1 response.

### Instrumentation

To measure eHealth literacy, an eHealth literacy scale [22] with 8 items was used. This variable was used to divide the groups into the low eHealth literacy group and the high eHealth literacy group using its average. The Cronbach α of eHealth literacy was .939 in this study. The sample items for eHealth literacy were “I know how to use digital technology to answer my health questions” and “I can tell the quality of health resources through digital technology.”

For TR [23], 16 items were used. The scale consisted of 4 subdimensions: optimism, innovativeness, discomfort, and insecurity. The sample items for TR included “New technologies contribute to a better quality of life” for optimism, “In general, I am among the first in my circle of friends to acquire new technology when it appears” for innovativeness, “Technical support lines are not helpful because they don’t explain things in terms I understand” for discomfort, and “People are too dependent on technology to do things for them” for insecurity.

PU, PEU, and attitude toward using digital health technology (ATDHT) are basic variables of the TAM. These variables were assessed by items developed by [7,17]. Finally, 5 items for continued usage intention [8] were used. The sample items were “Using Digital Health Technology would improve my exercise performance” for PU, “I would find it easy to get Digital Health Technology to do what I want it to do” for PEU, “Using Digital Health Technology is a good idea” for ATDHT, and “If I need to use Digital Health Technology, I will use it” for intention.

All responses were recorded on a 7-point Likert scale ranging from “strongly disagree” to “strongly agree.” First, the survey questionnaires were created in English because the original scales from the previous research were developed in English. The English version of the survey was then translated into Cantonese, which was the native language of the participants in this research, by an individual with a doctoral degree in sports management who also possessed a comprehensive understanding of public health literature and fluency in Cantonese and English. The Cantonese version was then back-translated into English by a different individual who possessed credentials similar to those of the individual who produced the Cantonese version. Finally, 10 potential participants who were above 60 years of age were recruited to check the questionnaire’s ease of use and clarity. As a result, the completed questionnaire was properly verified.

### Data Analysis

First, confirmatory factor analysis (CFA) was conducted to validate the posited relations between the observed variables and the underlying constructs in the measurement model. Various indexes, such as chi-square, the Steiger-Lind root mean square error of approximation (RMSEA), the Tucker–Lewis index (TLI), and the comparative fit index (CFI), were used to assess the absolute and comparative fit of the model. Second, composite reliability (CR), average variance extracted (AVE), and Cronbach α coefficients were calculated for the components of each measurement scale to check convergent validity, discriminant validity, and reliability. Finally, structural equation modeling (SEM) was applied to test the proposed hypotheses. The proposed model was also assessed by the same indexes used for the CFA.

### Ethical Considerations

The Research Ethics Committee of Hong Kong Baptist University in Hong Kong, China, approved the study (REC/20-21/0378).

## Results

### The Measurement Model

To assess the validity and reliability of the measurement model, we applied a 2-step structural equation modeling approach [24]. First, the measurement model was evaluated by CFA using AMOS 20. The results showed an acceptable fit to the data (*χ*^2^_532_=915.575; *P*<.001; TLI=0.935; CFI=.942; RMSEA=0.049). Regarding internal consistency, the range of CR and Cronbach α were from .806 to .937 and from .759 to .936, respectively. That is, the CRs and Cronbach α coefficients of all latent variables were higher than the criteria value of .7. This means that all latent variables had appropriate internal inconsistency.

Convergent validity is achieved by 2 criteria recommended by [25]. The CRs and AVEs for each latent variable should exceed thresholds of 0.7 and 0.5, respectively. The CRs (ranging from 0.806 to 0.937) and AVEs (ranging from 0.509 to 0.717) exceeded each threshold value, so all conditions for convergent validity were met. Discriminant validity is achieved if the square root of AVE for each latent variable exceeds all correlation coefficients among the latent variables. The range of correlation coefficients among variables was from −0.073 to 0.393. In this study, the minimum value of the square root of AVEs was 0.713. Because this was higher than the maximum value of the correlation coefficient (*r*=0.640), discriminant validity was also achieved. Table 3 shows the detailed results of the CFA, and Table 4 displays the basic statistics of the variables and the correlation coefficients among the variables.

**Table 3:** Confirmatory factor analysis for measurement model^a^.

	Range of χ^2^	Average variance extracted	Composite reliability	Cronbach α
Optimism	0.710-0.825	0.601	0.857	.856
Innovativeness	0.720-0.795	0.576	0.845	.843
Discomfort	0.521-0.756	0.509	0.806	.759
Insecurity	0.579-0.795	0.523	0.814	.787
Perceived usefulness	0.773-0.884	0.713	0.937	.936
Perceived ease of use	0.756-0.872	0.685	0.929	.928
Attitude toward using digital health technology	0.839-0.855	0.717	0.835	.853
Continued usage intention	0.567-0.845	0.541	0.852	.840

^a^*χ*^2^_532_=915.575, *P*<.001; Tucker–Lewis index=0.935, comparative fit index=0.942, root mean square error of approximation=0.049.

**Table 4:** Mean (SD) and correlation coefficients among variables.

	Optimism	Innovativeness	Discomfort	Insecurity	Perceived usefulness	Perceived ease of use	Attitude toward using DHT^a^	Continued usage intention	eHealth literacy
Optimism									
Innovativeness	.446**								
Discomfort	−.122**	−.037							
Insecurity	−.183**	−.142*	.449**						
Perceived usefulness	.470**	.465**	−.165**	−.165**					
Perceived ease of use	.481**	.622**	−.228**	−.174**	.638**				
Attitude toward using DHT	.491**	.413**	−.128*	−.169**	.572**	.551**			
Continued usage intention	.522**	.507**	−.061	−.129*	.640**	.597**	.593**		
eHealth literacy	.405**	.486**	−.070	−.077	.385**	.492**	.432**	.363**	
Mean (SD)	3.834 (0.661)	2.946 (0.856)	3.048 (0.696)	3.469 (0.748)	3.393 (0.731)	3.226 (0.739)	3.675 (0.710)	3.575 (0.639)	3.417 (0.805)
Skewness	−.275	−.136	−.276	−.496	−.400	−.098	−.648	−.138	−.587
Kurtosis	3.667	−3.303	3.456	3.166	3.219	3.043	4.055	3.307	3.589

^a^DHT: digital health technology.

^*^P<.05.

^**^P<.01.

### The Structural Model

Based on an appropriate measurement model, SEM was conducted to test the hypothetical causal relationships among the 8 latent variables. The results showed that our structural model had fairly acceptable fit indexes (*χ*^2^_542_=994.158; *P*<.001; TLI=0.924; CFI=931; RMSEA=0.052).

The hypothetical paths between TR and PU were not significant, except for the path from optimism to PU (H1-a). In the case of H2 regarding the effect of TR on PEU, all paths were significant except for the path from insecurity to PEU (H2-d). This means that H1 and H2 were partially supported. In other words, for adults over 60 years of age, optimism about DHT had a positive effect on PU (β=.265; *P*=.002) and PEU (β=.331; *P*<.000). Additionally, innovativeness had a positive effect on PEU (β=.559; *P*<.001). However, discomfort about DHT had a negative influence on PEU (β=−.397; *P*<.001).

The results of testing hypotheses H3 to H6 associated with the TAM were as follows. The path from PEU to PU was statistically significant (β=.513; *P*<.001). Additionally, 2 paths from PEU (β=.401; *P*<.001) and PU (β=.323; *P*<.001) to ATDHT were positively significant. Finally, ATDHT had a significant effect on older adults’ continued usage intention (β=.553; *P*<.001). This means that H3 to H6 were supported. Next, regarding hypothesis 7, our hypothesized model was independently analyzed for each group classified by the level of eHealth literacy. Four paths associated with the TAM were statistically significant in both groups. However, there was a difference in the paths in which TR affected PU and PEU. In the low eHealth literacy group, the negative influence of discomfort on PEU was found to be very powerful (β=−.646; *P*<.001). In the rest of the paths, the high eHealth literacy group (group B) tended to have a stronger influence on PU and PEU than the low literacy group (group A). The results of the hypothesis testing are shown in Table 5.

**Table 5:** Hypotheses testing^a^.

Hypothesis		Path	Estimate (*P* value) (n=306)	Group A (*P* value) (n=141)	Group B (*P* value) (n=165)
**H1**					
	a	Optimism to PU^b^	0.265 (.002)	0.192 (.12)	0.264 (.02)
	b	Innovativeness to PU	0.018 (.80)	−0.019 (.86)	0.113 (.31)
	c	Discomfort to PU	−0.013 (.90)	−0.307 (.23)	0.122 (.25)
	d	Insecurity to PU	−0.028 (.71)	0.109 (.58)	−0.103 (.18)
**H2**					
	a	Optimism to PEU^c^	0.331 (<.001)	0.187 (.10)	0.358 (.004)
	b	Innovativeness to PEU	0.559 (<.001)	0.456 (<.001)	0.678 (<.001)
	c	Discomfort to PEU	−0.397 (<.001)	−0.646 (.01)	−0.254 (.04)
	d	Insecurity to PEU	0.121 (.12)	0.363 (<.05)	−0.007 (.93)
H3		PU to PEU	0.513 (<.001)	0.567 (<.001)	0.417 (<.001)
H4		PU to ATDHT^d^	0.401 (<.001)	0.469 (<.001)	0.343 (<.001)
H5		PEU to ATDHT	0.323 (<.001)	0.298 (.01)	.310 (<.001)
H6		ATDHT to continued usage intention	0.553 (<.001)	0.495 (<.001)	.506 (<.001)

^a^*χ*^2^_542_=994.158; *P*<.001; Tucker–Lewis index=0.924; comparative fit index=0.931; root mean square error of approximation=0.052.

^b^PU: perceived usefulness.

^c^PEU: perceived ease of use.

^d^ATDHT: attitude toward using digital health technology.

## Discussion

### Principal Findings

By applying the TRAM to older adults in Hong Kong, this study (1) examined the factors affecting older adults’ intention to use DHT, (2) investigated their eHealth literacy level, and (3) explored the differences between participants with high and low levels of eHealth literacy regarding their DHT usage intentions. The findings are discussed below.

This study validated the TRAM in older adults in Hong Kong in terms of its construct validity, convergent validity, discriminant validity, and internal consistency. The results demonstrated that the TRAM is an appropriate and meaningful framework to predict older adults’ intentions to use DHT. Previous TRAM- or TAM-based studies mostly focused on health professionals’ acceptance of information and communication technology [26] and paid little attention to older adult users. The literature included insufficient knowledge about applying the TRAM to users’ attitudes and intentions toward health care and services. This study provides valuable information on this subject to this study and offers a more comprehensive understanding of older adults’s specific cognitive evaluations and decision-making processes for DHT usage.

The participants in the current research expressed a considerable extent of optimism and low discomfort toward DHT usage. Meanwhile, limited innovativeness and a degree of insecurity were demonstrated among this group of people as well. In terms of the relationships between TR variables and PU/PEU, for optimism, the study results indicated that optimistic participants were more likely to perceive DHT as useful and easy to use. This finding was consistent with previous research [20,21,27,28], which suggested that optimistic persons, including older persons, seem to confront DHT more positively, have more confidence in DHT usage, and pay less attention to the negative aspects of DHT. Inconsistent with the previous hypothesis, this study found that innovativeness was positively connected to PEU but not PU. A possible explanation is that older individuals’ innovativeness may benefit their experience with DHT usage and help them adapt to new IT, but whether DHT is useful depends on the function of the specific DHT tool. Similar results were found in the studies of [27] and [29]. It is notable that the effect of innovativeness on PEU was much stronger than that of optimism. This might be because healthy older adults are more likely to be an optimist yet with narrow innovativeness, according to the descriptive statistics of the variables in the current study. Another explanation is that older adults are not among the most frequent DHT users, and they must make an effort to adopt and adapt to new technologies. In this process, innovativeness is a stronger motivator than optimism [30]. Concerning discomfort, this study revealed that it negatively impacts PEU but not PU. This is likely because older adults may feel unfamiliar with many new technologies, and the perceived lack of control in applying DHT may make users more likely to perceive DHT as difficult to use. However, for older individuals, their primary concern with using DHT is staying healthy; if a helpful DHT tool is complex to use, older adults may still perceive it as useful. Such cases occurred not only among this group but also among health care practitioners [27], internet professionals [21], and young adults [20]. Surprisingly, this research found that insecurity appeared to be unrelated to either PU or PEU. This counterintuitive result conflicts with the results of other scholars [21,27,29]. One likely reason is that older adults rarely have the expertise to use DHT appropriately, and web-based misinformation causes older adults to distrust DHT to some extent [31]. This distrust may prevent users from using DHT more frequently [32]. It may be difficult for older adults to tell to what extent DHT is useful or easy to use if they use DHT superficially. This research provides additional evidence for the TR variables integrated into the TAM, demonstrating the applicability of its persistence among older individuals. It would be worthwhile for future studies to explore whether the relationships of the TRAM differ in different age groups.

For the relationships among the TAM variables, the results were as expected and aligned with previous studies [20,21,27,28]. First, a positive impact of PEU on PU was reported. This result confirmed the proposition of the TRAM that PEU is a precedent of users’ PU [33], especially for older individuals, because DHT is useless unless older adults are willing to use it. Second, PEU and PU were both found to positively contribute to attitudes toward using DHT, indicating that if older people perceive DHT as useful and easy to use, they are likely to have a positive attitude toward its use. In particular, the effect of PU on DHT is higher than that of PEU, which might be because users with health demands (ie, older adults) may place a higher value on PU than on PEU for DHT. Finally, participants with more positive attitudes were found to have a stronger intention to consistently use DHT. Attitude has long been seen as a predictor of behavioral intention [33,34], and the results of this study are in line with this understanding.

This research investigated eHealth literacy among older adults in Hong Kong and found that marital status and perceived socioeconomic status can influence their eHealth literacy status. Married older adults were more likely to receive social support for the use of DHT devices, which could result in high eHealth literacy. Older adults with higher income could have more experience with DHT devices, which may also help develop high eHealth literacy. The correlation analysis also showed that eHealth literacy was significantly connected with all the variables except discomfort and insecurity. This might be because the current measurement for eHealth literacy is developed on the basis of Web 1.0 technology, which is a web-based environment, while new technologies (eg, social networking services or mobile internet) have been applied in current DHT [32]. In that case, older adults may still be confused by new DHT and perceived discomfort and insecurity, even if they know how to obtain information from health-related websites. Moreover, this study explored the differences in the relationships of the TRAM between participants with high and low levels of eHealth literacy and confirmed the influences of eHealth literacy on the relationships. It was revealed that in the lower-level eHealth literacy group, the impact of optimism on PEU and PU was no longer significant, while in the higher-level group, this relationship remained significant. In addition, the influence of innovativeness on PEU was stronger for the higher-level group, and the influence of discomfort for the higher-level group was much weaker. In the TRAM, optimism and innovativeness are 2 driving forces for people’s acceptance of technology, and discomfort is an inhibitory factor. The study results demonstrated that higher-level eHealth literacy can promote the positive effect of TR variables and can be a protective factor against the negative impact. This finding emphasizes the important role of eHealth literacy in older adults’ acceptance of DHT, which provides insight for DHT developers and public health practitioners. The results can help DHT developers and public health practitioners better target DHT users of different ages and offer more effective assistance and guidelines for people with different eHealth literacy levels.

Our findings are meaningful for older adults because older individuals with eHealth literacy can increase their interest in health and knowledge of health care, which leads to confidence in DHT and a positive attitude toward DHT [35]. eHealth training can help enhance older adults’ skill level and ability to search, understand, and use DHT and improve negative emotions such as anxiety, fear, and stress that block DHT use [15]. It is expected that people could develop high eHealth literacy through well-designed eHealth training, and they could assess and choose accurate and necessary health information [36]. Therefore, appropriate theory-based eHealth literacy interventions based on high-quality research design should be implemented to effectively assist older adults in equipping themselves with the skills and knowledge necessary to benefit from eHealth resources [37].

### Limitations and Future Direction

Several limitations are recognized in this study. First, the participants of this study were recruited via a web-based survey, which means that the current study excluded older adults with less willingness to interact with IT. The generalizability of the research findings might be hindered by this sampling method. Related studies targeting people who are less willing to use IT are required because their health demands might be more pressing and they may receive less support than those who use IT. Second, the respondents of this study were older adults from Hong Kong. Considering the cultural specificity, the application of the study findings in other groups or areas should be further examined. Third, factors beyond the TRAM may also predict users’ intention of DHT usage, but they have not been considered in the current research. More in-depth studies that consider other potential influencers of DHT usage are desirable. Finally, the outcome variable of this study was DHT usage intention. Since a gap naturally exists between behavioral intentions and actual behavior and it is actual behavior that can influence individuals’ health status, studies that integrate behavior and identify more components in this relationship are warranted.

### Conclusions

This study tested the TRAM in older Hong Kong adults and explored the difference in the relationships of the TRAM between participants with high and low levels of eHealth literacy. The findings provided partial support for the hypotheses, emphasizing the impact of eHealth literacy on encouraging older adults to use DHT and obtain health benefits from it. This study also suggests providing assistance and guidelines for this population to narrow the aging-related technology gap and to further explore the associations of eHealth literacy, the TRAM, and actual behavior.

## Abbreviations

ATDHT: attitude toward using digital health technology
AVE: average variance extracted
CFA: confirmatory factor analysis
CFI: comparative fit index
CR: composite reliability
DHT: digital health technology
PEU: perceived ease of use
PU: perceived usefulness
RMSEA: Root Mean Square Error of Approximation
SEM: structural equation modeling
TAM: Technology Acceptance Model
TLI: Tucker–Lewis index
TR: technology readiness
TRAM: Technology Readiness and Acceptance Model
